# Modulation of *Arabidopsis* Flavonol Biosynthesis Genes by Cyst and Root-Knot Nematodes

**DOI:** 10.3390/plants9020253

**Published:** 2020-02-17

**Authors:** Noureddine Hamamouch, Brenda S. J. Winkel, Chunying Li, Eric L. Davis

**Affiliations:** 1 North Carolina State University, Department of Entomology & Plant Pathology, Raleigh, NC 27695, USA; cli2@ncsu.edu (C.L.); eric_davis@ncsu.edu (E.L.D.); 2 Laboratory of Biotechnology and Sustainable Development of Natural Resources, Polydisciplinary Faculty, University Sultan Moulay Slimane, Beni Mellal 23000, Morocco; 3 Virginia Tech, Department of Biological Sciences, Blacksburg, VA 24061, USA; winkel@vt.edu

**Keywords:** cyst nematode, flavonoids, *Heterodera schachtii*, *Meloidogyne incognita*, parasitism, root-knot nematode

## Abstract

Although it is well established that flavonoid synthesis is induced in diverse plant species during nematode parasitism, little is known about the regulation of genes controlling flavonol biosynthesis during the plant–nematode interaction. In this study, expression of the *Arabidopsis thaliana* flavonol-specific transcription factor, *AtMYB12*, the flavonol synthase genes, *AtFLS1*, *2*, *3*, *4*, and *5*, and the gene encoding the central flavonoid enzyme, chalcone synthase (*AtCHS*), were examined in plant roots during infection by *Heterodera schachtii* (sugar beet cyst) and *Meloidogyne incognita* (root-knot) nematodes. These experiments showed that *AtMYB12* was transiently upregulated at 9 dpi in syncytia associated with sugar beet cyst nematode infection and that an *Atmyb12*-deficient line was less susceptible to the parasite. This suggests that, rather than contributing to plant defense, this gene is essential for productive infection. However, the *AtCHS* and *AtFLS1* genes, which are controlled by AtMYB12, did not exhibit a similar transient increase, but rather were expressly downregulated in syncytia relative to adjacent uninfected root tissue. Genetic analyses further indicated that *AtFLS1* contributes to plant defense against Cyst nematode infection, while other *AtFLS* gene family members do not, consistent with prior reports that these other genes encode little or no enzyme activity. Together, these findings indicate a role of *AtMyb12* in promoting the early stages of Cyst nematode infection, while flavonols produced through the action of AtFLS1 are essential for plant defense. On the other hand, a transient induction of *AtMYB12* was not observed in galls produced during root-knot nematode infection, but this gene was instead substantially downregulated, starting at the 9 dpi sampling point, as were *AtCHS* and *AtFLS1.* In addition, both the *AtMYB12*- and *AtFLS1*-deficient lines were more susceptible to infection by this parasite. There was again little evidence for contributions from the other *AtFLS* gene family members, although an *AtFLS5*-deficient line appeared to be somewhat more susceptible to infection. Taken together, this study shows that sugar-beet cyst and root-knot nematodes modulate differently the genes involved in flavonol biosynthesis in order to successfully infect host roots and that AtFLS1 may be involved in the plant basal defense response against nematode infection.

## 1. Introduction

Plant parasitic nematodes are the causal agents of an estimated USD 100 billion in crop losses to the world-wide agro-economy annually, with Cyst *(Heterodera* and *Globodera* spp.) and root-knot (*Meloidogyne* spp.) nematodes causing the most extensive damage [1]. Plant infection by these nematodes involves the penetration of second-stage juveniles (J2) into host roots and migration through the root and subsequent intracellular migration (for cyst nematodes) or intercellular migration between the cortical cells to the vascular cylinder [2] until the favored site for feeding site induction is reached [3]. The nematodes then secrete an array of effectors into host cells through their stylet (a protrusible hollow mouth spear) to initiate the formation of specialized feeding structures known as syncytia for cyst nematodes and giant cells for root-knot nematodes [4,5,6]. These plant feeding cells, which are characterized by dense cytoplasm, enlarged multiple nuclei, a fragmented central vacuole and proliferation of organelles [3,5] act as the permanent source of nutrients for further nematode development. The feeding sites formed by cyst and root-knot nematodes have similarities in final phenotype but differ in ontogeny. Cell expansion with karyokinesis uncoupled from cytokinesis characterizes giant cells formed around the root-knot nematode head, while coordinated dissolution of walls adjacent to an initial syncytial cell give rise to a syncytium induced by cyst nematode [3]. The majority of effectors reported from cyst and root-knot nematodes differ, consistent with the differences observed in the parasitic process [6]. The secretion of nematode effectors is accompanied by an extensive alteration of gene expression in parasitized plant cells and roots including genes related to defense responses, cell-wall modifications, metabolic and signaling pathways, phytohormone balance, and the phenylpropanoid pathway [6,7,8,9,10,11,12]. Cyst and root knot nematodes induce the expression of different genes in *Arabidopsis thaliana* roots [7,12]. This is likely to be a reflection of the different modes of parasitism between cyst and root-knot nematodes as reflected in feeding cell ontogeny.

Flavonoids are natural products found in all plant species that are produced as part of the phenylpropanoid pathway [13]. In addition to ubiquitous pigments that play key roles in plant reproduction, seed dispersal, and UV protection [14,15], flavonoids are essential as chemical cues and defense molecules in interactions with microbes and insects [16,17,18,19,20]. There is also growing evidence that flavonoids contribute to defense against parasitic nematodes, although this remains poorly understood [21,22,23]. Flavonols represent the most abundant class of flavonoids and are also believed to be among the most ancient [24]. They possess potent free radical scavenging activity [25] and can have insecticidal properties [26]. In fact, soybean (*Glycine max*) genotype PI 227687, which accumulates the flavonol rutin, has been used widely in breeding programs as a source of insect resistance [27]. Flavonols are also involved in modulating auxin transport and signaling [28,29]. Several studies support a role of auxin in nematode feeding site formation of both cyst and root-knot nematode [30,31,32]. Moreover, flavonols have been shown to have a direct effect on chemotaxis, motility, and egg hatching of many nematode species [33]. Flavonols are synthesized from dihydroflavonols by a 2-oxoglutarate-dependent dioxygenase enzyme, flavonol synthase (FLS). *Arabidopsis* contains six *FLS* genes (*AtFLS1* to *AtFLS6*) located at three sites on chromosome 5 [34]. *AtFLS1*, *3*, and *5* encode full-length proteins while *AtFLS2*, *4*, *6* are considered pseudo-genes unlikely to contribute to flavonol synthase activity [34]. AtFLS1 has been shown to have substantial flavonol synthase activity, both in vivo and in vitro [34,35], while AtFLS2 exhibits a relatively small amount of activity [36] and AtFLS5 no detectable activity under any conditions tested to date.

The R2R3-MYB family of transcription factors participates in a variety of cellular processes, including development [37,38,39], signal transduction [40,41], cell division [42], secondary metabolism [43,44,45], and plant disease resistance [46]. Among members of the R2R3-MYB family in *Arabidopsis*, *AtMYB12* is a key regulator of FLS and chalcone synthase (CHS) gene expression [47,48,49]. CHS is the first enzyme in the flavonoid branch of the phenylpropanoid pathway and is encoded by the single-copy *AtCHS* gene in *Arabidopsis* [50]. Expression of *AtCHS* and *AtFLS1* is induced by overexpression of *AtMYB12* and substantially reduced in an *Atmyb12* T-DNA knockout, with corresponding changes in flavonol levels in *Arabidopsis* seedlings [48].

In the study presented here, quantitative real-time PCR was used to analyze the expression patterns of *AtMYB12* and the flavonoid biosynthetic genes, *AtCHS* and *AtFLS1-5* during *Arabidopsis* parasitism by the beet cyst nematode, *Heterodera schachtii* and the southern root-knot nematode, *Meloidogyne incognita*. Histochemical analyses were used to examine the cell-type expression of these genes in nematode-infected root tissues. In addition, the effects on nematode parasitism of *AtMYB12* overexpression and null mutations in *Atmyb12* and five *Atfls* genes were examined. Our hypothesis is that sugar beet cyst and root knot nematodes modulate the expression of genes involved in the biosynthesis of flavonols in order to successfully infect host roots.

## 2. Results

### 2.1. AtMYB12 Is Upregulated in Syncytia and Downregulated in Galls

**Figure 1:**
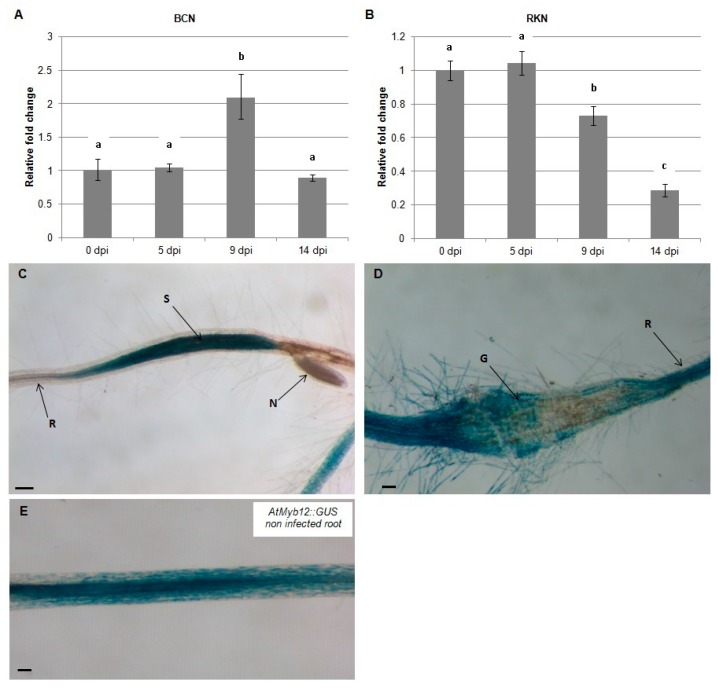
Temporal pattern of *Arabidopsis AtMYB12* gene expression during nematode infection. *AtMYB12* expression was quantified by qRT-PCR in roots of wild-type *Arabidopsis* plants at 0, 5, 9 and 14 dpi with the beet-cyst nematode, *H. schachtii* (BCN) (**A**) or the southern root-knot nematode *M. incognita* (RKN) (**B**). Histochemical localization of GUS activity directed by *AtMYB12p::GUS* fusion in *H. schachtii*-induced feeding sites (**C**) and in *M. incognita*-formed galls (**D**) at 9 dpi as compared to non-infected roots (**E**). G, gall; N, nematode; S, syncytium; R, *Arabidopsis* root. Quantitative expression analyses were normalized to the *Arabidopsis* Actin8 gene and presented relative to uninfected control tissue (baseline set at 1.0). Values are means ± SE (3 technical replicates) with different letters indicating significant differences between time points *p* < 0.05 (One-way ANOVA test). Similar results were obtained from two independent biological replicates. Scale bar = 200 μm (**C**), and 100 μm (**D**,**E**).

To test the effect of nematode infection on the flavonol transcriptional regulator, *AtMYB12*, quantitative real-time PCR (qRT-PCR) was used to quantify mRNA levels in *Arabidopsis* roots at 0, 5, 9, and 14 dpi with *H. schachtii* and *M. incognita.* During infection by *H. schachtii*, expression of *AtMYB12* increased at -9 dpi and returned to preinfection level by 14 dpi (Figure 1A). In contrast, during infection with *M. incognita*, expression of *AtMYB12* decreased over this time course, first detected at 9 dpi and continuing at 14 dpi (Figure 1B). These results indicate that *AtMYB12* is affected differently during *H. schachtii* and *M. incognita* infection.

To localize the expression of *AtMYB12* in nematode-infected roots, *AtMYB12p::GUS* plants were infected with *H. schachtii* and *M. incognita* and GUS expression was examined by histochemical staining at 9 dpi. The results of this experiment indicate that expression of *AtMYB12* is upregulated in syncytia formed by *H. schachtii* (Figure 1C) but strongly downregulated in galls generated by *M. incognita* (Figure 1D). These results further support the hypothesis that AtMYB12 mediates different responses to *H. schachtii* and *M. incognita* infection.

### 2.2. AtMYB12 Expression Promotes Sugar Beet Cyst Infection and May Contribute to Basal Defense Response to Root-Knot Nematode Infection

**Figure 2:**
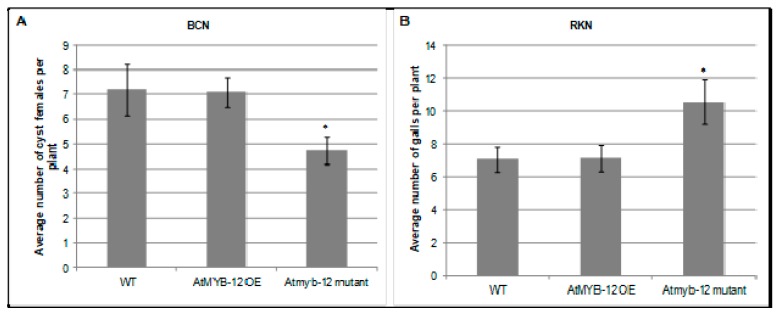
Role of *AtMYB12* in *H. schachtii* (**A**) and *M. incognita* (**B**) infection. Homozygous lines overexpressing *AtMYB12*, *Atmyb12* knock-out, and wild-type Col-0 (WT) plants were planted on modified Knop’s medium and 10 days-old seedlings were inoculated with approximately 60 surface sterilized J2 of either *H. schachtii* or *M. incognita*. Three weeks after inoculation, the number of cyst female nematodes (for suagr beet cyst nematode) per root system or galls (for southern root-knot nematode) were counted. Data are presented as means ± standard errors. Mean values significantly different from wild type (Col 0) as determined by *t* test (*p* < 0.05) are denoted by asterisks. Similar results were obtained from a second, independent biological replicate.

To assess the roles of AtMYB12 in nematode infection, *Arabidopsis* plants that either ectopically overexpress *AtMYB12* or carry a T-DNA insertion in the *Atmyb12* coding region were challenged with *H. schachtii* or *M. incognita* in six-well plates containing agar-based plant growth medium and the numbers of developing *H. schachtii* cyst females or *M. incognita* root galls in wild-type and mutant plants were counted 3–4 weeks post infection. No difference was observed in the number of *H. schachtii*-formed cysts or *M. incognita*-formed galls between plants over-expressing *AtMYB12* and wild-type *Arabidopsis* plants. However, the *Atmyb12* T-DNA mutant line was less susceptible to *H. schachtii* than were wild type plants, suggesting that the presence of AtMYB12 may promote *H. schachtii* parasitism (Figure 2). In contrast, *Atmyb12* T-DNA mutant plants were more susceptible to *M. incognita* than wild-type plants, suggesting that *AtMYB12* may contribute to basal defenses against *M. incognita*. These findings further suggest that *Myb12* upregulation in syncytia and downregulation in galls is induced by the nematodes in order to promote infection.

### 2.3. Expression of AtCHS and AtFLS1 Is Downregulated in Both Syncytia and Root Galls

**Figure 3:**
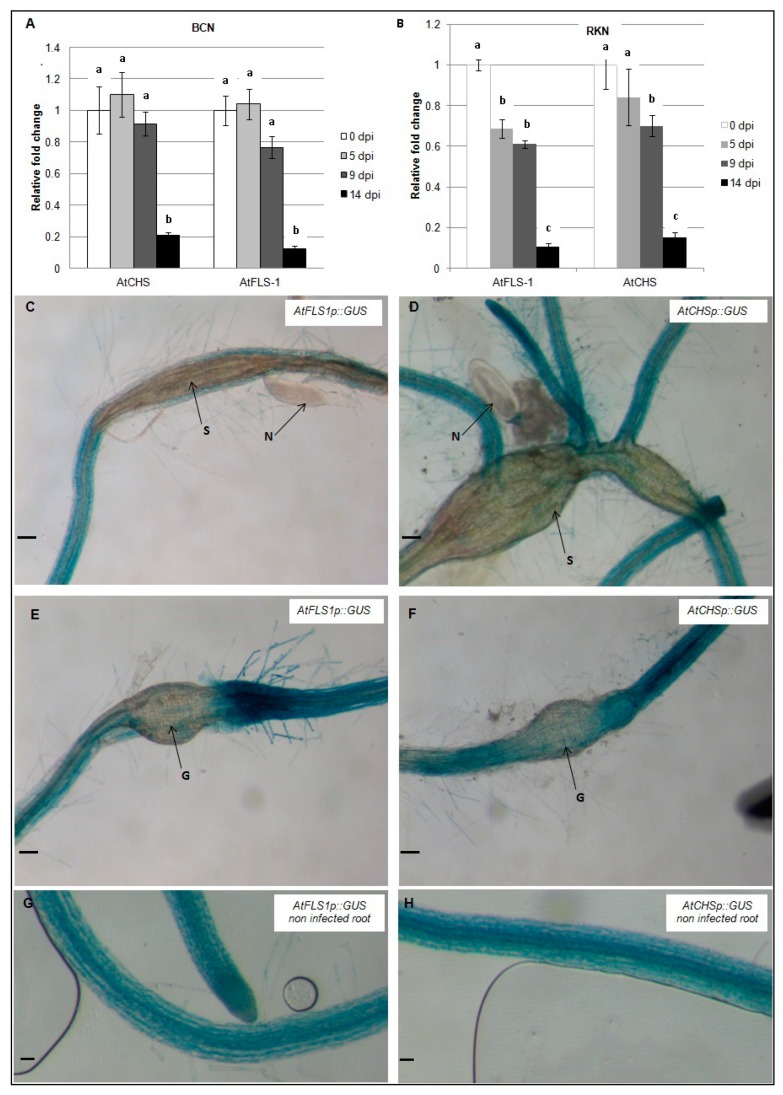
Temporal patterns of *AtCHS* and *AtFLS1* gene expression during nematode infection. *AtCHS* and *AtFLS1* gene expression was quantified by qRT-PCR in roots of wild-type *Arabidopsis* plants at 0, 5, 9 and 14 days post-infection (dpi) with the sugar beet cyst nematode, *H. schachtii* (**A**) or the southern root-knot nematode *M. incognita* (**B**). Histochemical localization of GUS activity directed by *AtCHSp::GUS* and *AtFLS1p::GUS* fusion in *H. schachtii*-induced syncytia (**C**,**D**) and in *M. incognita*-induced gall (**E**,**F**) at 9 dpi and in non-infected controls (**G**,**H**). G, gall; N, nematode; S, syncytium; R, *Arabidopsis* root. Expression was normalized to the Arabidopsis Actin8 gene and presented relative to expression at 0 dpi. Values are means ± SE (3 technical replicates) with different letters indicating significant differences between time points *p* < 0.05 (One-way ANOVA test). Similar results were obtained from a second, independent biological replicate. Scale bar = 200 μm (**C**,**D**,**G**,**H**), and 100 μm (**E**,**F**).

To examine the expression of *AtCHS* and *AtFLS1* during nematode infection, qRT-PCR was used to compare transcript abundance in *H. schachtii* and *M. incognita*-infected *Arabidopsis* roots at 0, 5, 9, and 14 dpi. Our results show that expression of *AtCHS* and *AtFLS1* was strongly reduced in *Arabidopsis* roots between 9–14 days following infection with *H. schachtii*. During infection with *M. incognita*, expression level of *AtFLS1* was observed by 5 dpi and 9 dpi, and at 14 dpi, it was strongly downregulated, while expression of *AtCHS* was reduced between 9–14 dpi (Figure 3A,B).

In addition, *AtCHSp::GUS* and *AtFLS1p::GUS* reporter genes were used to examine the expression level of *AtCHS* and *AtFLS1* at the nematodes feeding structures 9dpi. Histochemical analysis of GUS gene expression showed that expression of *AtCHS* and *AtFLS1* was strongly downregulated in syncytia and nematode-induced galls suggesting that the downregulation of these genes is important in nematode infection (Figure 3C–F).

### 2.4. Expression of AtFLS2 in Upregulated in H. schachtii-Induced Syncytia, but Downregulated in M. incognita-Induced Galls, While Expression of AtFLS5 Is Downregulated in Both.

**Figure 4:**
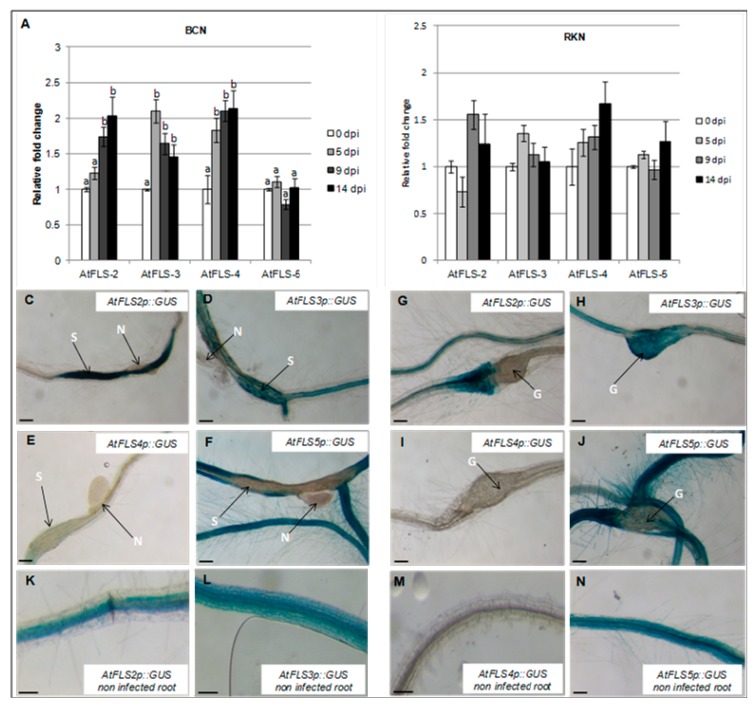
Temporal expression of *Arabidopsis* FLS gene family members during nematode infection. Expression of *AtFLS2*, *AtFLS3*, *AtFLS4*, and *AtFLS5* was quantified by qRT-PCR in roots of wild-type *Arabidopsis* plants at 0, 5, 9 and 14 dpi with the sugar beet cyst nematode, *H. schachtii* (**A**) of the southern root-knot nematode, *M. incognita* (**B**). Histochemical localization of GUS activity directed by *AtFLSp::GUS* in *H. schachtii*-induced syncytia (**C**–**F**) and in *M. incognita*-formed galls (**G**–**J**) at 9 dpi. (**K**–**N**) are non-infected Arabidopsis roots; G, galls; N, nematode; S, syncytium; R, *Arabidopsis* root. Expression was normalized to the Arabidopsis Actin8 gene and presented relative to expression at 0 dpi. Values are means ± SE (*n* = 3 technical replicates) with different letters indicating significant differences between time points *p* < 0.05 (One-way ANOVA test). Similar results were obtained from a second, independent biological replicate. Scale bar = 200 μm (**C**,**J**,**N**), and 150 μm (**K**–**M**).

The observed downregulation of *AtFLS1* in both nematode-induced syncytia and galls prompted us to examine the expression of four additional FLS genes to determine whether these might have a role in nematode infection. To this end, the abundance of *AtFLS2*, *AtFLS3*, *AtFLS4*, and *AtFLS5* transcripts in *Arabidopsis* whole roots at 0, 5, 9, and 14 dpi by *H. schachtii* and *M. incognita* was also measured using qRT-PCR. Our results showed that expression of *AtFLS2*, *AtFLS3*, and *AtFLS4* increased during *H. schachtii* infection, while expression of *AtFLS5* did not change (Figure 4A). In contrast, during infection with *M. incognita*, the expression of *AtFLS2*, *AtFLS3*, *AtFLS4*, and *AtFLS5* remained relatively unchanged or increased only slightly compared to non-infected roots (Figure 4B).

To visualize the expression *AtFLS2*, *AtFLS3*, *AtFLS4*, and *AtFLS5* at the nematode feeding sites, *Arabidopsis* plants expressing the *GUS* gene under the control of *AtFLS2*, *AtFLS3*, *AtFLS4*, and *AtFLS5* promoters were infected with *H. schachtii* or *M. incognita*, and *GUS* expression was visualized at the nematode-induced feeding sites. In *H. schachtii*-formed syncytia, *AtFLS2* was upregulated, suggesting that AtFLS2 may also play a role in syncytia formation and/or maintenance. The expression of *AtFLS5* was downregulated at 9 dpi (Figure 4C,F), while the expression of *AtFLS3* and *AtFLS4* did not change (Figure 4D,E). In *M. incognita*-formed galls, the expression of *AtFLS2* and *AtFLS5* was strongly downregulated (Figure 4G,J), while the expression of *AtFLS3* and *AtFLS4* did not change.

**Figure 5:**
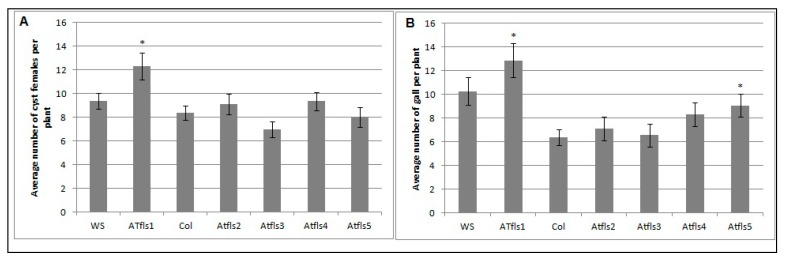
The effects of *Atfls* T-DNA mutations on *H. schachtii* (**A**) and *M. incoinita* (**B**) infection. *Atfls1*, *Atfls2*, *Atfls3*, *Atfls4*, and *Atfls5* knock-outs, two wild-type *Arabidopsis* plants; Col-0 and WS, were planted on modified Knop’s medium and 10-day-old seedlings were inoculated with approximately 60 surface sterilized J2 of either *H. schachtii* or *M. incognita*. Three weeks after inoculation, the number of cyst female nematodes (for sugar beet cyst nematode) per root system or galls (for southern root-knot nematode) were counted. Data are presented as means ± standard errors. Mean values significantly different from the wild type ecotype as determined by *t* test (*p* < 0.05). Similar results were obtained from a second, independent biological replicate.

The potential roles of *AtFLS1*, *AtFLS2*, *AtFLS3*, *AtFLS4*, and *AtFLS5* in *H. schachtii* and *M. incognita* infection of *Arabidopsis* were explored further using *Atfls* T-DNA knockout lines and wild type plants. The number of cyst females in *Atfls* mutant plants was not significantly different from those developed on the corresponding wild type ecotypes, with the exception of a significant increase in developed cyst nematodes on the *Atfls1* mutant (Figure 5A). These results suggest that AtFLS1 plays a role in *H. schachtii* infection of *Arabidopsis* roots, while the other four *AtFLS* genes have little or no effect. A similar significant increase was observed for the *Atfls1* and *Atfls5* mutants in the number of root-knot nematode galls, with the remainder of the *Atfls* mutants showing no difference in the number of galls compared to wild-type plants (Figure 5B). This suggests that both *AtFLS1* and *AtFLS5* may play a role in *M. incognita* infection.

### 2.5. Expression of AtFLS2, 3, 4, and 5 Is Dependent on AtMYB12

**Figure 6:**
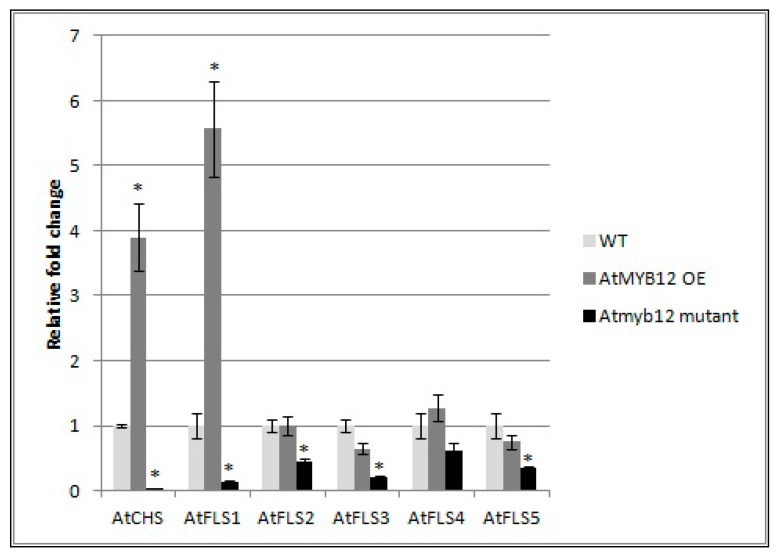
Effect of *AtMYB12* on the expression of *AtFLS* genes. Expression of *AtCHS* and *AtFLS1*, *AtFLS2*, *AtFLS3*, *AtFLS4*, and *AtFLS5* in plants overexpressing *AtMYB12*, *Atmyb122* knockout, and wild-type Col-0 (WT) seedlings using quantitative real-time PCR (qRT-PCR). The presented data are the mean fold changes ± standard errors in gene transcript levels relative to uninfected control tissue (baseline set at 1.0). Asterisks (*) indicate that the mean fold change is significantly different from 1.0 as determined by *t* test (*p* < 0.05). Similar results were obtained from a second, independent biological replicate.

To determine whether AtMYB also affects the expression of the other four *AtFLS* genes, qRT-PCR was used to compare the mRNA transcript levels of *AtFLS2*, *AtFLS3*, *AtFLS4*, and *AtFLS5* in *Arabidopsis* plants that over-express *AtMYB12* as well as in *Atmyb12* mutant plants. *AtFLS6*, which does not appear to be expressed, was not included in this analysis. We also measured the expression levels of *AtCHS* and *AtFLS1* transcripts as controls. Our results did confirm the finding of Mehrtens et al. [48], that the over-expression of *AtMYB12* induced expression of *AtCHS* and *AtFLS1*, while *Atmyb12* knockout reduced *AtCHS* and *AtFLS1* transcript levels to almost undetectable levels (Figure 6). In addition, while over-expression of *AtMYB12* had little or no effect on the transcript levels of the other four FLS genes, the *Atmyb12* knockout significantly reduced the expression of *AtFLS2*, *AtFLS3*, and *AtFLS5*, with a slight decrease also observed in expression of *AtFLS4* (Figure 6), indicating that *AtFLS2*, *3*, *4*, and *5* expression is dependent on AtMYB12.

## 3. Discussion

Flavonoids are natural plant products involved in diverse plant functions. A previous study using a flavonoid-specific stain showed that these compounds are produced in and around the developing syncytia of *H. schachtii* and galls of the European dagger nematode, *Xiphinema diversicaudatum*, respectively [22]. However, a detailed analysis of the expression of flavonoids biosynthesis genes during cyst and root-knot nematode parasitism has not previously been conducted. In this study, we examined the expression patterns of *R2R3-MYB12*, a flavonol-specific transcription factor; *AtCHS*, which encodes the first enzyme in the flavonoid pathway; and five flavonol synthase genes (*AtFLS1*, *AtFLS2*, *AtFLS3*, *AtFLS4*, and *AtFLS5*) during parasitism by *H. schachtii* and *M. incognita* and at the nematode-induced feeding sites. The role of these genes in nematode parasitism was investigated using T-DNA knockouts and over-expression lines.

Quantitative RT-PCR was first used to quantify *AtMYB12* transcripts in *H. schachtii* and *M. incognita*-infected *Arabidopsis* roots. It must be noted that only one reference gene (Actin 8) was used in qRT-PCR [51] and that small differences in expression observed among the data are subject to potential variations in reference expression [52,53]. *H. schachtii* appears to induce *AtMYB12* expression in *Arabidopsis* roots between 5–9 dpi. Similar to this finding, the genome-wide expression analysis of soybean roots infected with soybean cyst nematode showed that a soybean homolog of *AtMYB12* (BE0240360) is also upregulated in infected roots [11].

A histochemical analysis of *AtMYB12p::GUS* lines revealed that expression of *AtMYB12* increases in *H. schachtii*-formed syncytia, suggesting a role of this gene in syncytia formation. Previous studies on the expression of *AtMYB12* in syncytia have generated inconsistent results. Szakasits et al. [12] examined gene expression in *H. schachtii*-formed syncytia using Affymetrix GeneChips and showed that *AtMYB12* is downregulated in syncytia. In contrast, transcript profiling of cyst nematode feeding cells in soybean roots showed the soybean homolog of *AtMYB12* to be upregulated in syncytia [11]. Using the *AtMYB12p:GUS* lines offered the advantage of visualizing promoter activity within nematode-induced syncytia without the need for mRNA extraction, suggesting a role of this transcription factor in syncytia formation and/or maintenance.

The role of the transcription factor AtMYB12 in syncytia formation and development is still unclear, but AtMYB12 may induce the expression of downstream genes necessary for the formation and/or maintenance syncytia, such as genes involved in auxin transport. In fact, several auxin-responsive genes are modulated in tobacco plants over-expressing *AtMYB12*, including downregulation of an auxin efflux carrier [54,55]. Studies on the role of auxin in host-nematode interaction have suggested that changes in auxin levels, possibly achieved by a disruption of local auxin transport, may be important in feeding site formation of cyst nematodes [31,32,56]. A role for auxin in nematode feeding site development is supported by the finding that the cyst nematode effector protein 19C07 interacts specifically with the auxin influx transporter LAX3 [55], possibly to enhance auxin influx into host roots for syncytium development. The expression of *AtMYB12* in syncytia may be necessary to modulate host cell metabolism in order to increase the demands of the flux of amino-acids and macromolecules needed for nematode feeding. This latter hypothesis is supported by the increased expression of genes related to carbohydrates and lipid metabolism and the accumulation of amino-acids that have been reported to occur in tobacco plants over-expressing *AtMYB12* gene [54]. The reduction in the number of cyst females developing in *Arabidopsis Atmyb12* mutant plants compared to wild-type plants further supports the role of AtMYB12 in cyst nematode infection. However, overexpression of *AtMYB12* did not increase the susceptibility of *Arabidopsis* to *H. schachtii*.

On the other hand, infection with *M. incognita* decreased the expression of *AtMYB12* in the nematode-induced galls, suggesting that downregulation of *AtMYB12* may be necessary in establishing giant cells needed for nematode’s survival. The exact role of *AtMYB12* downregulation in nematode-induced galls in still unclear, but *M. incognita* may downregulate the expression of *AtMYB12* gene to limit expression of target genes that may interfere with giant cell formation and maintenance. However, the identity of these genes is still unknown and there is little information available on the genes, other than those involved in flavonoid biosynthesis, affected in *AtMYB12* knockout lines. The hypothesis that the downregulation of *AtMYB12* gene promotes giant cell formation is further supported by the finding that plants impaired in *AtMYB12* gene expression showed hyper-susceptibility to infection by *M. incognita* compared to wild-type plants. The observation that a single gene (*AtMYB12*) appears to play distinct roles in *H. schachtii* and *M. incognita* feeding site development is interesting but not surprising, as these two different nematodes secrete different effector proteins into host root cells [6,57,58], and regulate the expression of distinct genes for the formation and establishment of the feeding sites [7,8,12].

The expression of *AtMYB12* in *H. schachtii*-induced syncytia was not accompanied by an increase in *AtFLS1* and *AtCHS* gene expression, suggesting that, in syncytia, the expression of these two genes may not be under the control of AtMYB12. The observed downregulation of *AtFLS1*, which has substantial enzymatic activity among the *AtFLS* genes, in the feeding sites of both *H. schachtii* and *M. incognita* is surprising since flavonols are known to have potent free radical-scavenging activity [25] and it has been reported that ROS are produced in both syncytia and giant cells [59,60]. Downregulation of *AtFLS1* and *AtCHS* gene expression in syncytia has been previously reported in studies of syncytia transcriptome [12]. However, these two genes have not been identified to be differentially regulated in *M. incognita*-formed giant cell in *Arabidopsis* at least at very early infection stages [7].

Downregulation of key genes in flavonol biosynthesis (*CHS* and *FLS1*) at the nematode feeding structures may be necessary for feeding site formation and/or maintenance, perhaps related to the role of flavonols in modulating auxin transport [61]. A study on the effect of selected flavonoid compounds on the behavior of *M. incognita* showed that flavonols and chalcone, which are the products of *FLS1* and *CHS*, respectively, have inhibitory effects on motility and the hatching of nematodes [33]. The importance of *AtFLS1* downregulation in syncytia and giant cells formation was further supported by the hyper-susceptibility of an *AtFLS1*-deficient line. *Atfls1* T-DNA knockout lines appear also to be more susceptible to both *H. schachtii* and *M. incognita* compared to wild type plants. The downregulation of *AtFLS1* in nematode feeding sites, either directly or indirectly, may reduce potential defense responses from the flavonol pathway that could inhibit successful parasitism by nematodes.

## 4. Materials and Methods

### 4.1. Nematode Culture

Cyst nematodes of *H. schachtii* (BCN) and *M. incognita* (RKN) were cultured on roots of cabbage plants (*Brassica oleracea* var. capitata) and tomato plants (*Solanum lycopersicon* cv. Rutgers) grown in soil, respectively. Eggs of *H. schachtii* were collected from crushed cysts as previously described for cyst species [62], while eggs of *M. incognita* were extracted from tomato roots with sodium hypochlorite as previously described [63]. Eggs were hatched over water in Baermann pans at 28 °C for 48 h, and the hatched preparasitic J2 (pre-J2) were collected, surface-sterilized by incubation for 10 min in sterilization solution (0.004% mercuric chloride, 0.004% sodium azide and 0.002% Triton X-100), and rinsed three times with sterile distilled water.

### 4.2. Nematode Infection Assay and Data Collection

*Arabidopsis thaliana* (Columbia and Wassilewskija ecotypes) seeds were surface sterilized and transferred (one seed per well) into six-well culture plates (Falcon) containing 6 mL of sterile modified Knops medium [64] solidified with 0.8% Daishin agar (Brunschwig Chemie) as previously described [8]. Seeds on plates were placed in a 24 °C growth chamber under a 16 h light/8 h dark cycle for 2 weeks. Surface sterilized pre-J2 nematodes were suspended in 1.5% low-melting-point agarose to allow even distribution and to facilitate their movement into solid Knops medium. Plants were inoculated with approximately 60 J2 per plant and developed cysts (for sugar beet cyst nematodes) and galls (for root-knot nematodes) were counted 3–4 weeks post-infection, using a dissecting microscope. Nematode infection assays were conducted on two independent biological replicates, and similar results were obtained (the data of one experiment is presented). The means and standard errors of 18 replicates per treatment were calculated. Statistical differences were determined by the paired *t*-test with an alpha level of 0.05 using SAS software (Cary, NC, USA).

### 4.3. Plant Material

*Arabidopsis* lines containing *AtCHSp::GUS* and *AtMYB12P::GUS* were previously described [48] and were kindly provided by Dr. Bernd Weisshaar of the Max Planck Institute, Cologne, Germany. The *AtMYB12* T-DNA insertion line (Salk_046675C, insertion in third exon) and *AtMYB12* ectopic over-expression line (35S-*MYB12*, CS9603; [48] were obtained from the Arabidopsis Biological Resource Center (ABRC, The Ohio State University, Columbus, OH, USA). *AtCHSp::GUS*, *AtFLSp::GUS*, and T-DNA insertion lines for *AtFLS1*, *2*, *3*, *4*, and *5* were previously described [34,65]. The T-DNA lines were AJ588535 (insertion in the 5′ untranslated region of *AtFLS1*), SALK_076420 (*AtFLS1* promoter), GABI 429B10 (second intron of *AtFLS2*), SALK_050041 (third exon of *AtFLS3*), SALK_002309 (third exon of *AtFLS4*), and GABI 317E12 (first intron of *AtFLS5*).

### 4.4. Histochemical Localization of GUS

*Arabidopsis* roots infected with *H. schachtii* and *M. incognita* were excised 9 dpi. Histochemical staining for GUS expression was performed at 37 °C for 4 h using X-Gluc solution [(0.1 M NaH_2_PO_4_, 10 mM EDTA, 0.5 mM each of K_3_Fe(CN)_6_ and K_4_F_2_(CN)_6,_ 3H_2_O, 0.1% Triton X-100 and 1 mg/mL 5-bromo-4-chloro-3-indolyl-beta-D-glucuronic acid (cyclohexylammonium salt) (Gold Biotechnology, St. Louis, MO, USA)], and then mounted onto glass slides. Samples were examined using a Nikon SMZ 800 stereo microscope, and images were captured with a SPOT 2 digital camera (Diagnostic Instruments, Inc., Sterling Heights, MI, USA).

### 4.5. RNA Isolation and Quantitative RT-PCR 

Total RNA was isolated from whole roots of six *Arabidopsis* plants using the RNeasy Plant Mini Kit (QIAgen, Valencia, CA, USA) following the manufacturer’s instructions. Prior to quantitative RT-PCR, total RNA was treated with RNase-free DNase I (Ambion, Austin, TX, USA) to eliminate any contaminating genomic DNA. First-strand cDNA was synthesized from 2–3 µg of total RNA using SuperScript-II RT (Invitrogen, Carlsbad, CA, USA) and oligo-dT_18_ primers following the manufacturer’s instructions.

All reactions were performed in a DNA Engine Mx3000P (Agilent Technologies, Santa Clara, CA, USA). Each 20 μL qRT-PCR reaction contained 1X Brilliant II SYBR Green qPCR Master Mix (Agilent Technologies, Santa Clara, CA, USA), 5 μL cDNA template and 5 μM each forward and reverse primers (Table 1). The PCR cycling parameters were set at 95 °C for 10 min followed by 40 cycles of 95 °C for 15 s, 60 °C for 1 min, and 72 °C for 1 min. At the completion of each reaction, dissociation melt curve analyses (60–90 °C every 0.5 °C for 1 s) were conducted to discount the effects of primer-dimer formation and contamination. The qRT-PCR reactions were performed in three technical triplicates. Using the 2^−ΔΔ*C*_T_^ method [66], the data are presented as the fold change in gene expression normalized against the endogenous *Arabidopsis actin 8* gene (At1g49240), and presented relative to expression at 0 dpi. qRT-PCR experiments were conducted on two independent biological replicates and similar results were obtained (the data of one experiment was presented). Values are means ± SE (*n* = 3 technical replicates). One-way ANOVA with a Student–Newman–Keuls post hoc test was used to determine significant differences between means.

## Figures and Tables

**Table 1:** Primers used in Real-time PCR.

Gene Name	Locus Name	Primers	Target Size (bp)
*AtMYB12*	At2g47460	F; 5′-AACCAAGGGAATCTCGACTGTCT-3′ R; 5′-CCCAATCGATAAACTCATCCGT-3′	109
*AtCHS*	At5g13930	F; 5′-CGCATCACCAACAGTGAACAC-3′ R; 5′-TCCTCCGTCAGATGCATGTG-3′	94
*ATFLS1*	At5g08640	F; 5′-CCGTCGTCGATCTAAGCGAT-3′ R; 5′-CGTCGGAATCCCGTGGT-3′	107
*ATFLS2*	At5G63580	F; 5′-TCTTATGGCCAAGACGATCC-3′ R; 5′-GAAAAATGCCCCACTCTTCA-3′	101
*ATFLS3*	At5G63590	F; 5′-CGACGCGGAGTATACCACTT-3′ R; 5′-TATCCATCTTCGCCCTATGC-3′	107
*ATFLS4*	At5G63595	F; 5′-GGGATCCCAACCGAACTAAT-3′ R; 5′-TCTCTTTGGAGTTCGCTGGT-3′	109
*ATFLS5*	At5G63600	F; 5′-AAGCCTTCAAGGACGAACAA-3′ R; 5′-CTTTAACCTCCCGTTGGTCA-3′	107
*Actin 8*	At1g49240	F; 5′-GATGGAGACCTCGAAAACCA-3′ R; 5′-AAAAGGACTTCTGGGCACCT-3′	108
